# Mature iPSC-derived astrocytes of an ALS/FTD patient carrying the TDP43^*A90V*^ mutation display a mild reactive state and release polyP toxic to motoneurons

**DOI:** 10.3389/fcell.2023.1226604

**Published:** 2023-07-28

**Authors:** Fabiola Rojas, Rodrigo Aguilar, Sandra Almeida, Elsa Fritz, Daniela Corvalán, Estibaliz Ampuero, Sebastián Abarzúa, Polett Garcés, Armando Amaro, Iván Diaz, Cristian Arredondo, Nicole Cortes, Mario Sanchez, Constanza Mercado, Lorena Varela-Nallar, Fen-Biao Gao, Martin Montecino, Brigitte van Zundert

**Affiliations:** ^1^ Faculty of Medicine and Faculty of Life Sciences, Institute of Biomedical Sciences (ICB), Universidad Andres Bello, Santiago, Chile; ^2^ Department of Neurology, University of Massachusetts Chan Medical School (UMMS), Worcester, MA, United States; ^3^ Department of Biology, Faculty of Chemistry and Biology, Universidad de Santiago, Santiago, Chile; ^4^ Millennium Institute Center for Genome Regulation CRG, Santiago, Chile

**Keywords:** ALS, FTD, TARDBP, TDP-43, astrocytes, non-cell autonomous, reactive, polyP

## Abstract

Astrocytes play a critical role in the maintenance of a healthy central nervous system and astrocyte dysfunction has been implicated in various neurodegenerative disorders, including amyotrophic lateral sclerosis (ALS) and frontotemporal dementia (FTD). There is compelling evidence that mouse and human ALS and ALS/FTD astrocytes can reduce the number of healthy wild-type motoneurons (MNs) in co-cultures or after treatment with astrocyte conditioned media (ACM), independently of their genotype. A growing number of studies have shown that soluble toxic factor(s) in the ACM cause non-cell autonomous MN death, including our recent identification of inorganic polyphosphate (polyP) that is excessively released from mouse primary astrocytes (*SOD1*, *TARDBP*, and *C9ORF72*) and human induced pluripotent stem cells (iPSC)-derived astrocytes (*TARDBP*) to kill MNs. However, others have reported that astrocytes carrying mutant TDP43 do not produce detectable MN toxicity. This controversy is likely to arise from the findings that human iPSC-derived astrocytes exhibit a rather immature and/or reactive phenotype in a number of studies. Here, we have succeeded in generating a highly homogenous population of functional quiescent mature astrocytes from control subject iPSCs. Using identical conditions, we also generated mature astrocytes from an ALS/FTD patient carrying the TDP43^*A90V*^ mutation. These mutant TDP43 patient-derived astrocytes exhibit key pathological hallmarks, including enhanced cytoplasmic TDP-43 and polyP levels. Additionally, mutant TDP43 astrocytes displayed a mild reactive signature and an aberrant function as they were unable to promote synaptogenesis of hippocampal neurons. The polyP-dependent neurotoxic nature of the TDP43^*A90V*^ mutation was further confirmed as neutralization of polyP in ACM derived from mutant TDP43 astrocytes prevented MN death. Our results establish that human astrocytes carrying the TDP43^*A90V*^ mutation exhibit a cell-autonomous pathological signature, hence providing an experimental model to decipher the molecular mechanisms underlying the generation of the neurotoxic phenotype.

## Introduction

Amyotrophic lateral sclerosis (ALS) and frontotemporal dementia (FTD) form a continuous spectrum of aggressive neurodegenerative diseases, affecting primarily motor and cortical frontotemporal neurons. Although most cases of ALS are sporadic (sALS), about 10% are familial ALS (fALS), and transmitted as dominant traits. There is a score of genes carrying specific mutations that have been grouped according to their ability to cause fALS, including superoxide dismutase 1 (*SOD1*, incl. SOD1^*G93A*^ and SOD1^*A4V*^), trans-active response DNA-binding protein 43 (*TARDBP* encoding TDP-43, incl. TDP43^*A315T*^ and TDP43^*A90V*^), FUS RNA binding protein (*FUS*), valosin-containing protein (*VCP*), and *C9ORF72* (characterized by an intronic hexanucleotide expansion) (DeJesus-Hernandez et al., 2011; Al-Chalabi and Hardiman, 2013; Sreedharan and Brown, 2013; Renton et al., 2014; Taylor et al., 2016; van Zundert and Brown, 2017). Individuals harboring mutations in *TARDBP* and C9ORF72 can develop ALS, FTD or both (ALS/FTD) and hence exhibit motor dysfunction as well as cognitive impairment (Ling et al., 2013; Ferrari et al., 2019). Currently, there are no effective treatments for ALS or ALS/FTD.

Evidence from animal models indicate that astrocytes contribute to motoneuron (MN) degeneration and are important players in the onset and progression of ALS (Clement et al., 2003; Lepore et al., 2008; Yamanaka et al., 2008; Ilieva et al., 2009; Papadeas et al., 2011; Wang et al., 2011; van Zundert et al., 2012; Qian et al., 2017; Tong et al., 2013; Ramírez-Jarquín et al., 2017). Astrocytic non-cell autonomous processes have also been demonstrated in multiple mouse and human cell-based assays (van Harten et al., 2021; Stoklund Dittlau and Van Den Bosch, 2023). Thus, primary ALS and ALS/FTD astrocytes derived from transgenic mouse models harboring pathogenic gene mutations in *SOD1, TARDBP* and *C9ORF72* reduce the number of healthy wild-type MNs in co-cultures or after application of astrocyte conditioned media (ACM) (Vargas et al., 2006; Di Giorgio et al., 2007; Nagai et al., 2007; Fritz et al., 2013; Re et al., 2014; Rojas et al., 2014; Ikiz et al., 2015; Rojas et al., 2015; Jury et al., 2020; Mishra et al., 2020; Arredondo et al., 2022). Similarly, a loss of MNs is observed with human primary astrocytes, or human astrocytes generated from astrocyte precursors (APCs) or neural progenitor cells (NPCs) isolated from the central nervous system (CNS) from postmortem patients with SOD1 mutations (Haidet-Phillips et al., 2011) and sALS patients (Haidet-Phillips et al., 2011; Re et al., 2014).

While these *in vitro* studies using mouse and human glia cells have been critical to demonstrate the fundamental role of astrocytes in ALS, the mechanism(s) underlying their neurotoxic properties have not been elucidated, hampering the development of astrocyte-targeted therapeutic strategies. It has become increasingly clear that astrocytes are remarkably heterogeneous and plastic cells. Hence, their phenotype properties are influenced by the developmental stage as well as the environment present at the surrounding CNS region, including interactions with other cells such like MNs and microglia (Cahoy et al., 2008; Phatnani et al., 2013; Escartin et al., 2021). Astrocytes can also become reactive in response to pathological conditions in the surrounding tissue (i.e., CNS disease or injury) or to experimental manipulations (Escartin et al., 2021). Regarding the latter, cultured primary astrocytes–even healthy control astrocytes dissected from neonatal tissue and grown in the presence of serum–display a reactive phenotype compared to astrocytes *in vivo* (Cahoy et al., 2008; Phatnani et al., 2013). The phenotype of astrocytes, APCs or NPCs derived from postmortem fALS and sALS patients may be additionally influenced by the inflammatory and necrotic environment. Together, *in vitro* studies using primary astrocytes (and APCs or NPCs) have not allowed the identification of the innate ALS astrocyte signature, limiting the development of disease-ameliorating therapies.

Studies with human astrocytes generated from fibroblasts that have been reprogrammed to become induced pluripotent stem cells (iPSCs) or induced NPCs (iNPCs; termed also i-astrocytes) can help avoiding many of the problems observed with primary astrocytes and therefore have become valuable tools for disease modeling and therapeutic testing. Importantly, reduced MN survival has been found using human iPSC- or iNPC-derived astrocytes carrying mutations in *SOD1* (Meyer et al., 2014; Almad et al., 2022; Gomes et al., 2022), *C9ORF72* (Meyer et al., 2014; Birger et al., 2019; Varcianna et al., 2019), *VCP* (Hall et al., 2017) or using astrocytes derived from sALS patients (Meyer et al., 2014; Almad et al., 2022; Gomes et al., 2022). Additional studies using human iPSC-based models indicate that astrocytes harboring mutations in *C9ORF72* (Zhao et al., 2020) or *FUS* (Stoklund Dittlau et al., 2023) caused a milder MN toxicity, characterized by MN pathophysiology (but not death), whereas astrocytes carrying a mutation in *TARDBP* (TDP43^*M337V*^) did not lead to detectable MN toxicity (Serio et al., 2013).

Generation of ALS ACM has increased the potential for identifying toxic factor(s) that cause non-cell autonomous MN death. We recently showed that inorganic polyphosphate (polyP), a ubiquitous, negatively charged biopolymer, is a critical toxic factor in non-cell-autonomous MN degeneration and a potential therapeutic target for ALS/FTD (Arredondo et al., 2022). Thus, it was found that mouse (*SOD1*, *TARDBP*, and *C9ORF72*) and human (*TARDBP*; mutTDP43^*A90V*^) iPSC-derived astrocytes display elevated levels of intracellular polyP. We also showed that polyP is excessively released by mouse and human ALS and ALS/FTD astrocytes and, importantly, demonstrated by gain- and loss-of-function approaches, that polyP causes non-cell autonomous death of MNs (Arredondo et al., 2022). Recent analyses of iPSC cell-based models further suggest that toxic effects of human astrocytes on MN survival and neurite outgrowth may also be the result of an enhanced secretion of inflammatory cytokines from reactive FUS-ALS astrocytes (Stoklund Dittlau et al., 2022) as well as increased release of ATP released through the opening of Cx43 hemichannels that are upregulated in fALS SOD1 and sALS astrocytes (Almad et al., 2022). Conversely, loss of neuron-supportive mechanisms has also been proposed to contribute to MN death and impaired neurite network maintenance (Stoklund Dittlau and Van Den Bosch, 2023). Specifically, it was found that ACM and/or extracellular vesicles released from diverse human ALS astrocytes subtypes was toxic to MNs due to decreased levels of miR-494-3p in C9ORF72-ALS (Varcianna et al., 2019), reduced levels of miR-146a in SOD1-ALS and sALS (Gomes et al., 2022), and lower concentrations of anti-oxidant proteins in C9ORF72-ALS (Birger et al., 2019).

Different ALS and ALS/FTD astrocyte subtypes could be linked to specific mechanisms during astrocyte-mediated MN death. Moreover, MN death may result from the generation of immature and/or reactive astrocytes. To confirm the generation of mature astrocyte cultures, most teams perform immunostaining to detect well-established astrocyte markers, including glial fibrillary acidic protein (GFAP), S100 calcium-binding protein β (S100β) and aldehyde dehydrogenase-1 L1 (ALDH1L1). While GFAP is a widely-used astrocyte marker, it is also indicative of astrocyte reactivity (high GFAP expression relates to a reactive state) and of the astrocyte maturation stage (low GAP expression relates to a mature astrocyte state) (Cahoy et al., 2008; Roybon et al., 2013; Escartin et al., 2021). Thus, *in vitro* and *in vivo* studies using mouse and human astrocytes indicate that elevated GFAP expression in cultured astrocytes reflects an immature phenotype (Roybon et al., 2013 and references herein). Furthermore, studies using human iPSCs/iNPCs-derived control and ALS astrocytes (identified by robust expression of S100β, ALDH1L1 or NF1A) show that even control astrocytes display a strong expression of GFAP (Serio et al., 2013; Meyer et al., 2014; Hall et al., 2017; Birger et al., 2019; Zhao et al., 2020; Gomes et al., 2022). These results therefore suggest that ALS astrocytes (generated under comparable experimental conditions) possess a rather immature and/or reactive phenotype, thereby unable to accurately mimic the disease condition.

Here, we report the generation of functional quiescent mature astrocytes from control subject-derived iPSCs. Using identical conditions, we also generated mutTDP43 mature astrocytes from ALS/FTD patient-derived iPSCs carrying the TDP43^*A90V*^ mutation. These patient-derived mature astrocytes exhibit key pathological hallmarks, including enhanced cytoplasmic TDP43 and polyP levels, a mild reactive state and neurotoxic properties. Importantly, we show that MN death was prevented by neutralizing polyP in mutTDP43-ACM.

## Materials and methods

### Animals for primary spinal cord neuronal cultures

All protocols involving rodents (including rat spinal cord cultures; see below) were carried out according to the NIH and ARRIVE guidelines and were approved by the Ethical and Bio-security Committees of Universidad Andres Bello. Sprague-Dawley rats, to obtain embryonic ventral spinal cord cultures (E14), were originally obtained from the Pontifical Catholic University of Chile (Santiago) and maintained and bred at the animal facility of Universidad Andres Bello.

### Astrocytic differentiation from iPSCs

Fully reprogrammed iPSC lines were previously generated from skin biopsies by retroviral transduction using the four Yamanaka factors (OCT4, SOX2, KLF4, and cMYC) (Zhang et al., 2013). Biopsies were derived from an ALS/FTD patient (75 years old male) carrying an *A90V* mutation in TARDBP (TDP43^*A90V*^; line FTD36L10) and from a healthy subject (56 years old female, termed control; line FTD37L20), a family member without mutations (Zhang et al., 2013). Maintenance of iPSCs and differentiation to NPCs, astrocyte precursor cells and finally mature astrocytes were performed as previously described (Almeida et al., 2012; Zhang et al., 2013; Arredondo et al., 2022). Briefly, iPSCs were maintained in feeder-free conditions using mTeSR1 medium (STEMCELL Technologies, Cat. # 85850). EBs were generated in EB Differentiation Medium KnockOut DMEM/F12 media (Gibco, Cat. #12660-012) supplemented with 10% KnockOut serum replacement (Gibco, Cat. #10828-028), 1x GlutaMax (Gibco, Cat. #35050-061), 1x NEAA (Gibco, Cat. #11140-050), and 2-mercaptoethanol (Sigma-Aldrich, Cat. #M3148)] and maintained in suspension for 1 week. Rosette-shaped neuroepithelial cells were obtained after plating the EBs in plates coated with poly-L-ornithine (Sigma-Aldrich, Cat. #P4957) and laminin (Sigma-Aldrich, Cat. #L2020) and grown for 1 week in Neural Induction Medium [KnockOut DMEM/F12 supplemented with N2 (Gibco, Cat. #17502-048), NEAA, 2 mg/mL heparin (Sigma-Aldrich, Cat. #H3149), and 10 ng/mL βFGF (Gibco, Cat. #PHG0021)]. Rosettes were manually isolated under the microscope, re-plated in plates pre-treated with Matrigel (Corning, Cat. #354277) and grown for one more week in Neural Expansion Medium [Neurobasal supplemented with Glutamax, NEAA, B-27 (Gibco, Cat. #17504-044), and βFGF]. Rosettes were disaggregated using Accutase Cell Detachment Solution (EMD Millipore, Cat. # SCR005) to generate a monolayer culture of Neural Progenitor Cells (NPCs). NPCs were differentiated to astrocyte precursor cells by culturing for 2 weeks in astrocyte precursor medium [KO DMEM/F12, 1x StemPro NSCs Supplement (Gibco, Cat. #A10508-01), 10 ng/mL Activin A (Gibco, Cat. #PHC9564), 10 ng/mL Heregulin 1β (R&D Systems, Cat. #377-HB-050), 200 ng/mL IGF1 (R&D System, Cat. #P291-G1-200), 20 ng/mL βFGF, 20 ng/mL EGF (Gibco, Cat. #PHG0311), 1x GlutaMAX] (Shaltouki et al., 2013). Then, precursor cells were incubated for two additional weeks in astrocyte maturation and maintenance medium [DMEM/F12, 2% B27, 10 ng/mL Heregulin, 5 ng/mL BMP2 (BioVision, Cat. #4577-50) and 2 ng/mL CNTF (R&D Systems, Cat. #257-NT-010)] (Malik et al., 2014).

### Alternative differentiation media

Differentiation from astrocyte precursor cells was also tested using alternative medium 1 [Neurobasal, 5 ng/mL CNTF, 10 ng/mL BMP2, 8 ng/mL FGF, 1% FBS, 1x B27, 1x NEAA, 1x GlutaMAX, 50 μg/mL Penn-Strep] (Shaltouki et al., 2013) or using alternative medium 2 [Neurobasal, 100 nM TSA (first 48 h), 500 nM 5-azaC (first 48 h), 20 ng/mL BMP2, 1x B27, 1x GlutaMAX, 50 μg/mL Penn-Strep, 10 ng/mL LIF] (Majumder et al., 2013).

### Collection of astrocyte conditioned media (ACM)

At the final step of culturing astrocytes (or APCs), the cultures were maintained under media conditions that enable later to study the viability of MNs in rat primary spinal cord cultures. Briefly, precursor or mature astrocyte cultures were incubated in Neuronal Growth Medium [MEM (Gibco, Cat. #11090-073) supplemented with 25% Neurobasal media (Gibco, Cat. #21103-049), 1% N2 supplement (Gibco, Cat. #17502-048), 1% L-glutamine (Gibco, Cat. #25030-081), 1% penicillin-streptomycin (Gibco, Cat. #15140-122), 2% horse serum (Gibco, Cat. #15060-114; lot 1517711) and 1% sodium pyruvate (Gibco, Cat. #11360-070)]. After 7 days, conditioned medium was collected and supplemented to 4.5 mg/mL D-glucose, filtered, and stored at −80°C. The control-ACM and TDP43-ACM was used to evaluate MN survival (diluted 6-fold). Using spinal cord cultures, we verified that neither control nor ALS conditioned medium did change the expression pattern of specific canonical markers for key developmental stages, including MAP2, GFAP, S100b, Nestin y CD44 (data not shown).

### Cell labeling of specific markers for human iPSCs and differentiations

Precursor or mature astrocyte cultures were fixed at 2, 14, 19, 21 and 28 DIV with 4% paraformaldehyde (Sigma-Aldrich, Cat. #P6148), and immunostained with antibodies against CD44, GFAP, S100β, ALDH1L1, Cx43, EAAT2, AQP4, Islet-1, MAP2, Nestin and Iba1, as described in Table 1. Antibody binding was visualized with the appropriate fluorescent secondary antibodies as described in Table 2. Immunolabeled APCs or astrocytes were documented on an inverted Nikon Eclipse Ti-U microscope equipped with a SPOT Pursuit™ USB Camera CCD (14-bit), Epi-FL illuminator, mercury lamp, and Sutter Smart-Shutter with a lambda SC controller. Cells were photographed using a ×40 objective; positive cells were counted offline within five randomly chosen fields, and the percentage of positive cells compared to the total number of TO-PRO (Invitrogen, Cat. #T3605) positive cells was calculated.

**TABLE 1:** List of antibodies used in this study.

Primary antibody	Brand	Catalog number	Host	Reacts to	Dilution
AQP4	Abcam	ab46182	Rabbit polyclonal	Hum Ms Rat	1:200
ALDH1L1	NeuroMab	75-140	Mouse monoclonal	Hum Ms Rat	1:50
CD44	BD Biosciences	550392	Mouse monoclonal	Hum Ms	1:1000
Cx43	Invitrogen	13-8300	Mouse monoclonal	Hum Ms Rat	1:200
EAAT2	Invitrogen	PA5-17099	Rabbit polyclonal	Hum Ms Rat	1:50
GFAP	Invitrogen	MA5-15086	Mouse monoclonal	Hum Ms Rat	1:600
GFAP	Dako	z0334	Rabbit polyclonal	Hum Ms Rat	1:1000
Islet-1	Abcam	ab20670	Rabbit polyclonal	Hum Ms Rat	1:300
MAP2	Sigma	M9942	Mouse monoclonal	Hum Ms Rat	1:1000
MAP2	Invitrogen	OSM00030W	Rabbit polyclonal	Ms Rat	1:600
Nestin	Millipore	MAB5326	Mouse monoclonal	Hum no rodent	1:500
S100β	Abcam	ab4066	Mouse monoclonal	Hum Ms Rat	1:400
S100β	Dako	Z0311	Rabbit polyclonal	Hum	1:1000
SMI32	Covance/Biolegend	SMI-32R/801701	Mouse monoclonal	Mammalian	1:600
SOX1	Abcam	ab22572	Rabbit polyclonal	Hum Ms	1:100
TDP43	Encor	MCA-3H8	Mouse monoclonal	Hum Ms Rat	1:150
Iba1	Santa Cruz	sc-32725	Mouse monoclonal	Hum Ms Rat	1:150

**TABLE 2:** List of fluorescent secondary antibodies used in this study.

Secondary antibody	Brand	Catalog number	Host	Reacts to	Dilution
Alexa Fluor 488 Gt antiMs	Invitrogen	A-11029	Goat	Mouse (Ms)	1:500
Alexa Fluor 546 Gt antiMs	Invitrogen	A-11003	Goat	Mouse (Ms)	1:500
Alexa Fluor 633 Gt antiMs	Invitrogen	A-21052	Goat	Mouse (Ms)	1:500
Alexa Fluor 488 Gt antiRb	Invitrogen	A-11034	Goat	Rabbit (Rb)	1:500
Alexa Fluor 546 Gt antiRb	Invitrogen	A-10035	Goat	Rabbit (Rb)	1:500
Alexa Fluor 633 Gt antiRb	Invitrogen	A-21070	Goat	Rabbit (Rb)	1:500

### Primary spinal cord neuronal cultures

To analyze ACM toxicity of mouse or human IPSC-derived astrocytes towards MNs, investigators have used primary rodent (rat and mouse) spinal cord cultures, mouse ESC-derived MNs and human ESC-derived MNs. Mixed astrocyte-MN species cultures (rat, mice, human) do not appear to induce any side effects and typically 40%–50% MN loss is detected in all studies using ALS-ACM or ALS astrocytes (e.g., Di Giorgio et al., 2007; Nagai et al., 2007; Fritz et al., 2013; Meyer et al., 2014; Re et al., 2014; Arredondo et al., 2022). As in our previous studies, we have used rat spinal cord cultures to obtain high quality MNs, from which we have extensive functional data (patch-clamp and calcium fluxes) and identified fatal pathogenic signaling pathways induced by ALS/FTD-ACM (Fritz et al., 2013; Rojas et al., 2014; Rojas et al., 2015; Arredondo et al., 2022). Briefly, pregnant Sprague–Dawley rats were deeply anesthetized with CO_2_, and primary spinal cultures were prepared from E14 pups Next, whole spinal cords were excised and placed in ice-cold PBS (Gibco, Cat. #14185-052) containing 50 μg/mL penicillin/streptomycin (Gibco, Cat. #15070-063). The dorsal part of the spinal cord was removed using a small razor blade, and the ventral cord was minced and enzymatically treated by incubating in pre-warmed PBS containing 0.25% trypsin (Gibco/Invitrogen, Cat. #15090-046) for 20 min at 37°C. Cells were transferred to a 15 mL tube containing neuronal growth media containing 70% MEM (Gibco, Cat. #11090-073), 25% Neurobasal media (Gibco, Cat. #21103-049), 1% N2 supplement (Gibco, Cat. #17502-048), 1% L-glutamine (Gibco, Cat. #25030-081), 1% penicillin-streptomycin (Gibco, Cat. #15070-063), 2% horse serum (Hyclone/Cytiva, Cat. #SH30074.03; Gibco, Cat. #15060-114) and 100 mM sodium pyruvate (Gibco, Cat. #11360-070); they were precipitated, transferred to a new 15 mL tube containing 2 mL of growth media, re-suspended by mechanical agitation through fire-polished glass Pasteur pipettes of different tip diameters, and counted; 1 × 10^6^ cells were plated on freshly prepared poly-L- lysine-coated 24-wellplates (1 mg/mL; 30.000–70.000 MW; Sigma-Aldrich, Cat. #P2636). Cells were cultured for 7 days at 37°C under 5% CO_2_ and supplemented with 45 μg/mL chick hind limb muscle extract (Sepulveda et al., 2010); the media was refreshed every 3 days.

### Pharmacological treatment in culture

G4-PAMAM-NH2 (Sigma-Aldrich, Cat. #412449) was dissolved and used to final concentration of 1 μg/mL. Stock solution was stored at −20°C.

### Sandwich co-culture

The sandwich co-culture was performed by placing two 12 mm glass coverslips in each well of a 6-well culture plate per condition. Briefly, paraffin wax was heated to ∼100°C, an aliquot of ∼2 mL was taken with a Pasteur glass pipette and three small drops were applied near the outer edge of each coverslip at roughly equal distances from each other (Boraso and Viviani, 2011; Jones et al., 2012). The coverslips were sterilized by UV irradiation for 30 min, followed by the culture of primary spinal cord neurons as described previously. The ventral spinal cord neurons were seeded in the coverslips with paraffin dots. After 4 DIV forceps were used to lift the edge of a coated glass coverslips plated with ventral spinal cord neurons in the precursor or mature astrocyte cultures with ventral spinal cord neuron media. Primary ventral spinal cord neurons faced the precursor or mature astrocyte culture to allow the exchange of factor(s). The sandwich co-culture was maintained in a humidified 5% CO_2_ incubator at 37°C without changing the media for 3 days.

### Cell labeling and counting of MNs in spinal cord cultures

MNs and interneurons were immunolabeled and counted as previously described (Sepulveda et al., 2010; Fritz et al., 2013; Rojas et al., 2014; Rojas et al., 2015). Briefly, primary spinal cultures were fixed at 7 DIV with 4% paraformaldehyde, and immunostained with an antibody against MAP2 (Table 1) to label all neurons (interneurons plus MNs) and with the SMI-32 antibody (Table 1) to reveal the presence of unphosphorylated neurofilament-H, which is expressed specifically in MNs in spinal cord cultures (Urushitanietal., 2006; Nagai et al., 2007; Arredondo et al., 2022); antibody binding was visualized with the appropriate fluorescent secondary antibodies (Table 2). Our wild-type primary spinal cord cultures typically contain at least 6%–10% MNs until 12 DIV (Sepulveda et al., 2010). Immunolabeled neurons were documented on an inverted Nikon Eclipse Ti-U microscope equipped with a SPOTPursuit™ USB Camera CCD (14-bit), Epi-FL illuminator, mercury lamp, and Sutter Smart-Shutter with a lambda SC controller. Cells were photographed using a ×20 objective; MAP2 and SMI-32 positive neurons were counted offline within 20 randomly chosen fields, and the percentage of SMI-32-positive MNs within the total number of MAP2-positive cells was calculated.

### Primary hippocampal neuronal cultures

Cultures of hippocampal neurons were prepared from embryonic day (E) 18 Sprague-Dawley rat fetuses as previously described (Ampuero et al., 2017; Bustos et al., 2017). Briefly, E18-pregnant rats were deeply anesthetized with CO_2_ and hippocampi were excised and placed into ice-cold PBS containing 50 μg/mL penicillin/streptomycin. The extracts were minced and incubated for 20 min at 37°C in pre-warmed PBS containing 0.25% trypsin and then transferred to a tube containing Dulbecco’s modified Eagle’s medium supplemented with 10% horse serum and 100 U/mL penicillin/streptomycin. Then, cells were resuspended by mechanical agitation through fire-polished glass Pasteur pipettes of decreasing diameters. Cells were counted and plated on freshly prepared poly-L-lysine-coated 24 well plates (1 mg/mL; Sigma-Aldrich, Cat. #P2636). Plating media was replaced by growth media Neurobasal (Gibco/Invitrogen, Cat. #21103-049) supplemented with B27 (Gibco/Invitrogen, Cat. #17504044), 2 mM L-glutamine (Gibco/Invitrogen, Cat. #25030-081), 100 U/mL penicillin/streptomycin (Gibco/Invitrogen, Cat. # 15070-063). On day 2, hippocampal neurons were treated with 2 μM cytosine arabinoside for 24 h. Next, growth media was replaced with half of new media every 2–3 days.

### Immunofluorescence and cluster analysis

Immunofluorescence assays were performed as previously described (Bustos et al., 2014; Ampuero et al., 2017). Hippocampal cells were incubated from 7 to 12 DIV with ACM from mutTDP43-ACM and control-ACM (diluted 1:4) with replenishment of the ACM every 2 days. For immunofluorescence, hippocampal cultures were rinsed twice in ice-cold PBS and fixed for 20 min in a freshly prepared solution of 4% PFA with 4% sucrose in PBS. Then, the cells were rinsed three times in cold PBS and permeabilized for 5 min with 0.2% Triton X-100 in PBS. After that, for both conditions the cells were rinsed in ice-cold PBS and incubated in 1% BSA plus 3% donkey serum in PBS for 30 min at RT, followed by an overnight incubation at 4°C with primary antibodies. Primary antibodies used were PSD-95 (1:500; UC Davis/NIH NeuroMab Facility, Cat. #75-028), Synapsin (1:1000, Santa Cruz Biotechnology, Cat. #sc-20780), MAP2 (1:400, Santa Cruz Biotechnology, Cat. #sc-20172). Cells were washed three times with PBS, then incubated with the corresponding Alexa-conjugated secondary antibodies (Table 2) for 30 min at 37°C. Coverslips were mounted with Fluoromount-G (Electron Microscopy Sciences, Cat. #17984-25) and analyzed by confocal laser microscopy (with a ×60 oil objective; NA = 1.35, Olympus FV1000). Images were analyzed using NIH ImageJ software. Triple immunofluorescent images were captured by multitracking imaging of each channel independently, to eliminate possible crosstalk between the different fluorochromes. For cluster quantification, 8-bit images of maximal projections were analyzed using plugins of the Fiji software. All clusters with a minimal arbitrary gray level pixel intensity of 40 (out of 255) and size larger than 0.02 μm^2^ were analyzed on primary dendrites of MAP2-positive hippocampal neurons. For each condition, at least three dendrites per neuron (17 neurons), obtained from 3 independent experiments, were analyzed.

### Quantification and statistical analysis

Statistical analyses were performed using GraphPad Prism 6.01 software. Student’s t-test was performed when two populations were examined. One-way ANOVA followed by the Bonferroni *post hoc* was utilized when making multiple (three or more) comparisons. In all figures, the data is reported as mean ± S.E.M.; *p < 0.05, **p < 0.01, ***p < 0.001. In all experiments (except in Figure 2A), each condition was replicated in three independent differentiation cultures.

## Results

### Differentiation and characterization of human mature astrocytes derived from control and mutTDP43 iPSCs

**FIGURE 1:**
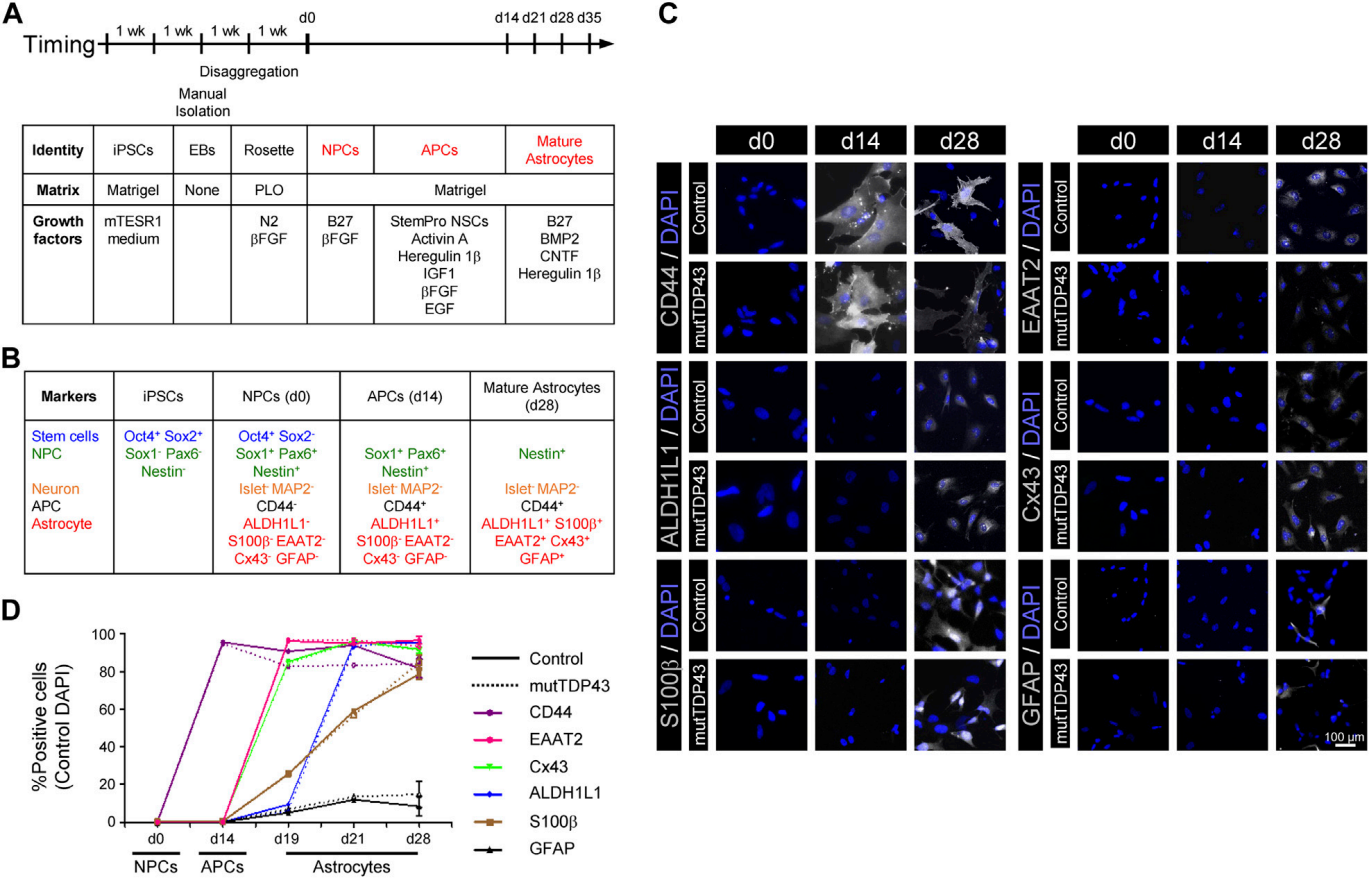
Differentiation and characterization of mature astrocytes generated from control subject-derived iPSCs and mutTDP43 patient-derived iPSCs. **(A)** Overview of the differentiation process of mature astrocytes from human iPSCs with principal growth factors and matrix types used. **(B)** Canonical markers used to validate the differentiation process. **(C)** Representative fluorescence microscopy images of each of the three differentiation stages: NPCs (0 days), APCs (14 days) and mature astrocytes (28 days). Staining was performed with APC (CD44) and with the following mature astrocyte markers (white): ALDH1L1, S100β, EAAT2, Cx43 and GFAP. Cell nuclei were stained with DAPI (blue). **(D)** Quantification of the percentage of positive cells for the different APC and mature astrocytes (MA) markers for a control subject (solid line) and an ALS/FTD patient carrying the TDP43^*A90V*^ mutation, termed mutTDP43 (dashed line). Data was obtained from three independent differentiations starting from NPCs, with at least 15 cells analyzed per condition in each experiment. Student’s *t*-test analysis revealed no significant difference (*p* > 0.05) between control and patient samples for any NPC, APC and mature astrocyte cultures at any given time point. For all data, see Table in Supplementary Figure S3.

We analyzed fully reprogrammed iPSC lines from skin biopsies derived from an ALS/FTD subject, carrying an *A90V* mutation in *TARDBP* (termed mutTDP43) and from a control subject, a healthy family member (termed control) (Zhang et al., 2013). From the human iPSCs, we generated embryoid bodies (EBs) (containing the three germ layers), rosettes (neuroepithelial cells), and then neural progenitor cells (NPCs) in a process that took around 4 weeks (Figures 1A, B; Supplementary Figures S1A, B). The identity and karyotype (which was normal) of the control and mutTDP43 iPSCs were previously reported (Zhang et al., 2013). Here, we verified the presence of the heterozygotic mutation in RNA samples obtained from control and mutTDP43 NPCs (Supplementary Figure S1C). Using immunofluorescence staining assays for SOX1 and Nestin on isolated and disaggregated rosettes, we also confirmed the neural nature of our NPCs (Supplementary Figures S1D, E). To further verify the lineage transition from iPSCs to NPCs, we examined by qRT-PCR the expression of canonical markers of pluripotency and NPCs of control subjects. As expected, *OCT4* (a master pluripotency transcription factor; Pan et al., 2002) was highly expressed in iPSCs and strongly reduced in NPCs, *SOX2* (a master regulator for both pluripotency and neural identity; Favaro et al., 2009) was present in both stages albeit at lower levels in NPCs, and *PAX6* (a critical transcription factor for neural differentiation; Gajović et al., 1998) was strongly expressed in NPCs but nearly detectable in iPSCs (Supplementary Figure S1E). The same analysis using patient cells revealed that the expression of pluripotency and NPC markers was similar to that in control subject and mutTDP43 cultures (Supplementary Figures S1E, F), indicating that both iPSC lines display a comparable stem cell differentiation process to the neural lineage.

Our next goal was to develop an efficient, reproducible and scalable method to generate, from human NPCs, sufficient mature astrocytes for the production of ACM. Although NPCs are potential sources of unlimited quantities of astrocytes, the generation of large, homogeneous, and mature populations of this type of glia cells has been challenging because NPCs, and similar to the embryonic brain in early stages, have a strong neurogenic bias, with limited astrogenesis capacity that is only acquired in late development (Hirabayashi and Gotoh, 2010). To examine whether large and homogeneous populations of APCs were obtained from control subject NPCs, we tested the expression of CD44 (Moretto and Lotti, 1993; Alfei et al., 1999) in three different protocols using previously described media formulations (Majumder et al., 2013; Shaltouki et al., 2013). Starting with NPCs (day 0, d0), it was found that two of the protocols led to an efficient generation of CD44^+^ APCs in 2 weeks (d14; see Figure 1C, D; Supplementary Figure S2). However, only one protocol (method 2; Shaltouki et al., 2013) resulted in APCs that maintained a high proliferation capacity, and hence enabling to generate scalable cultures of mature astrocytes.

Next, APCs were supplemented with B27, BMP2, CNTF and Heregulin 1β in the absence of serum (Figure 1A; Malik et al., 2014). Using immunostaining assays, it was determined that by d28 the cultures were characterized by a high percentage of cells expressing the mature astrocytic markers ALDH1L1 (>95%), S100β (>80%), EAAT2 (equivalent to rodent GLT1; >95%) and Cx43 (>90%), concomitant with a low percentage of cells expressing GFAP (<15%) (Figure 1B–D; Supplementary Figure S3). Based on detailed analysis of the expression pattern of these astrocyte markers during development and postnatal maturation in rodent and human cultures (Roybon et al., 2013), newly established criteria indicate that a mature astrocytic phenotype is reached at d28 of differentiation *in vitro* (see discussion). Immunostaining analyses for the neuronal markers MAP2 and Islet-1 revealed that neurons were only sparsely (<3%) detected in the cultures (Supplementary Figure S3). Immunostaining assays for the microglial marker Iba1 revealed that the cultures are essentially devoid of microglial cells (data not shown). Together, these data indicate that astrocytes characterized by a high degree of maturity and purity can be generated from iPSCs.

To determine whether the conversion of NPCs to mature astrocytes was similarly efficient for control and mutTDP43 cells, we assessed the expression of mature astrocytic markers -along with the NPC markers Nestin and the APC marker CD44 in both cultures at days d0, d14, d19, d21 and d28. These immunostaining assays indicated that control and mutTDP43 cultures exhibit nearly identical differentiation patterns at NPC, APC and mature astrocyte stages (Figures 1C, D; Supplementary Figure S3).

**FIGURE 2:**
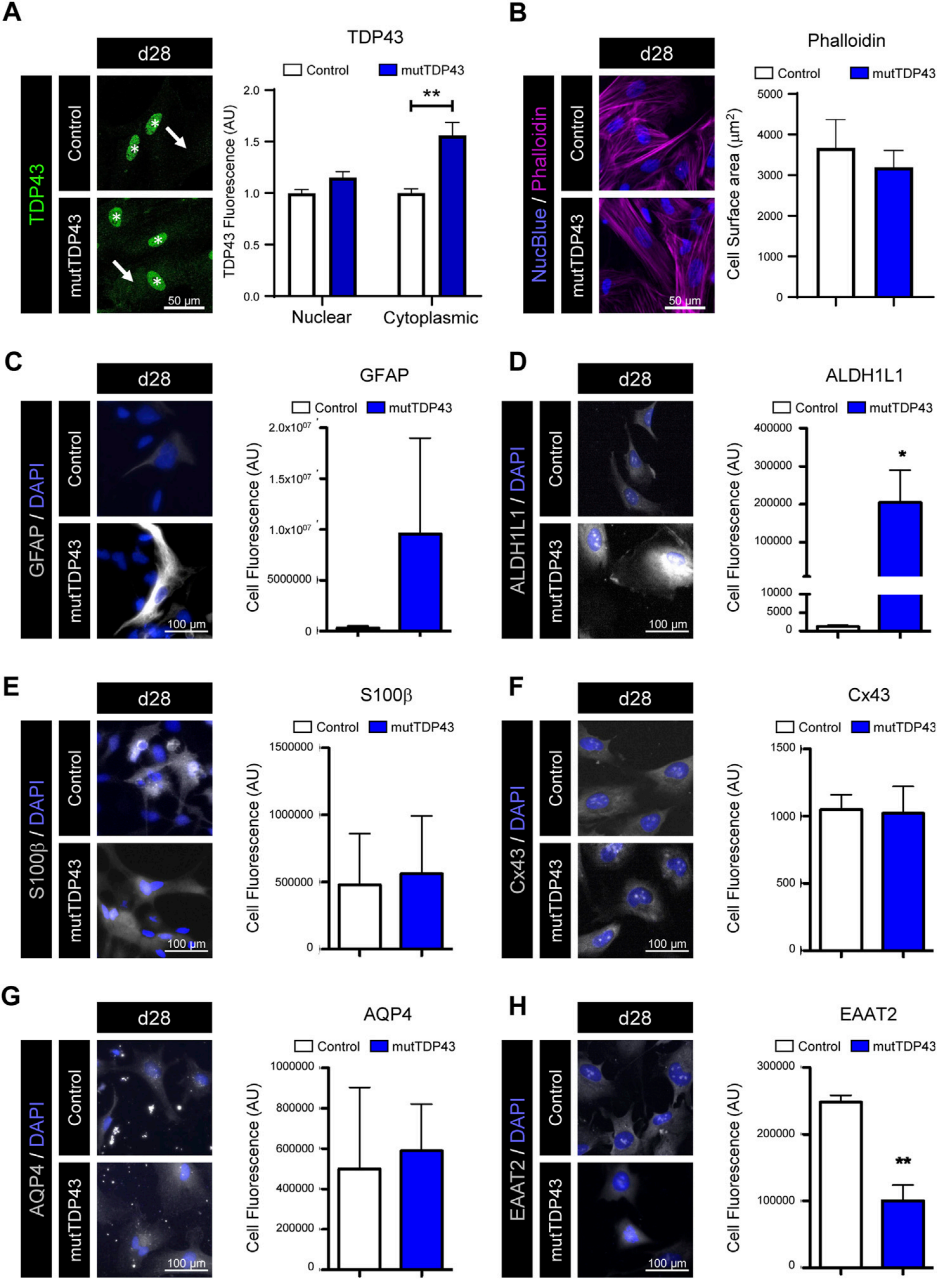
Mature mutTDP43 patient-derived astrocytes display mild manifestations of an intrinsic reactive inflammatory state. **(A–H)** Representative immunofluorescence images of control and mutTDP43 mature astrocytes at day 28 (d28) with nuclear marker NucBlue **(A–B)** or DAPI **(C–H)** (blue). **(A)** Immunostaining with antibodies against TDP-43 (green) showing robust staining in nuclei (asterisk) in both control and mutTDP43 astrocytes and only a mild enrichment of TDP-43 in the cytoplasm of mutTDP43 astrocytes (arrow). Graphs show relative TDP43 fluorescence intensity to the control condition and expressed as arbitrary units (AU) from 10 random isolated cells analyzed per condition from 1 differentiation. *P* < 0.05 unpaired Student’s *t*-test patient versus control. **(B)** Immunostaining with phalloidin-actin, showing flat polygonal fibroblast-like morphologies in both control subject- and patient-derived astrocytes. Graph shows cell surface area of control and mutTDP43 astrocytes (using phalloidin immunostaining) analyzing 8-10 random isolated cells per condition from 1 differentiation. *P* > 0.05 unpaired Student’s *t*-test patient versus control. **(C–H)** Representative images showing control and mutTDP43 mature astrocytes immunostained with antibodies for the following astrocyte markers (white) that are implicated in a reactive inflammatory phenotype **(C)** GFAP, **(D)** ALDH1L1, **(E)** S100β, **(F)** Cx43, **(G)** AQP4 and **(H)** EAAT2. The graphs show mean ± S.E.M. of the average fluorescent intensity of the cell soma and expressed as AU. *p < 0.05, **p < 0.01 unpaired Student’s *t*-test patient versus control with 15 cells analyzed per condition in each experiment from three independent differentiations.

We next investigated whether the mutTDP43 mature astrocytes recapitulate a critical pathological hallmark of ALS/FTD by displaying an abnormal TDP43 localization, including nuclear depletion and cytoplasmic accumulation/deposition in neurons and glia cells (Neumann et al., 2006; Geser et al., 2008; Zhang et al., 2013; Gregory et al., 2020; Baughn et al., 2023). iPSC-derived astrocytes carrying an ALS-causing TDP43 mutation (mutTDP^*M337V*^), showed TDP43 accumulation in the cytoplasm without detectable aggregation and without significant loss of nuclear TDP43 levels (Serio et al., 2013). In agreement with that study, our confocal imaging and quantification analyses revealed a ∼1.5-fold increased TDP43 enrichment in the cytoplasm of human mutTDP43 mature astrocytes (mutTDP^*A90V*^) compared to control subject astrocytes (Figure 2A). This cytoplasmic staining in mutTDP43 astrocytes was rather diffuse and not accompanied by detectable TDP43 aggregates and/or inclusions. Immunostaining analysis of the same cells also revealed that nuclear TDP43 levels were similar in mutTDP43 and control mature astrocytes.

### Mature mutTDP43 patient-derived astrocytes display mild signals of an intrinsic reactive state

To get insights into the reactive/quiescent state of our generated control and mutTDP43 astrocytes, we analyzed the morphology and expression of markers associated with reactivity and inflammation (e.g., GFAP). Previous studies showed that a quiescent state of cultured mouse and human astrocytes is characterized by a polygonal fibroblast-like morphology and low expression of GFAP, whereas a reactive inflammatory phenotype of astrocytes is marked by a process-bearing (or stellate) morphology and increased GFAP immunostaining (Cassina et al., 2005; Tchieu et al., 2019). Analysis of the cell morphology of our mature astrocytes by phalloidin-actin dye staining revealed that both control and mutTDP43 mature astrocytes exhibit a rather flat polygonal fibroblast-like morphology and similar cell surface area (Figure 2B). While we found that the percentage of d28 mature astrocytes positive for GFAP was low (<15%) in both control and mutTDP43 patient cultures (Figures 1C, D), in some mature mutTDP43 astrocytes the GFAP immunostaining intensity was strongly enriched without significantly changing the average fluorescence intensity relative to control mature astrocytes (Figure 2C). To further evaluate the quiescent/inflammatory phenotype of our mature astrocytes, we analyzed the immunofluorescence level of astrocyte markers that are either elevated (i.e., ALDH1L1, S100β, Cx43, aquaporin-4 [AQP4]; Figures 2D–G) or decreased (i.e., EAAT2; Figure 2H) in reactive astrocytes (Rothstein et al., 1995; Roybon et al., 2013; Götz, 2015; Barbar et al., 2020). Hence, it was determined that in mutTDP43 mature astrocytes the fluorescence intensity of the markers ALDH1L1 and EAAT2 was significantly increased and reduced, respectively, whereas the signal levels of CD44, S100β and AQP4 remained unchanged, relative to control subject mature astrocytes. Together, these results indicate that mature astrocytes generated from control subject iPSCs adopt a phenotype reminiscent of quiescent astrocytes, whereas mature mutTDP43 patient-derived astrocytes display mild manifestations of an intrinsic reactive inflammatory state.

### Mature mutTDP43 patient-derived astrocytes are unable to promote synapse maturation of hippocampal neurons

**FIGURE 3:**
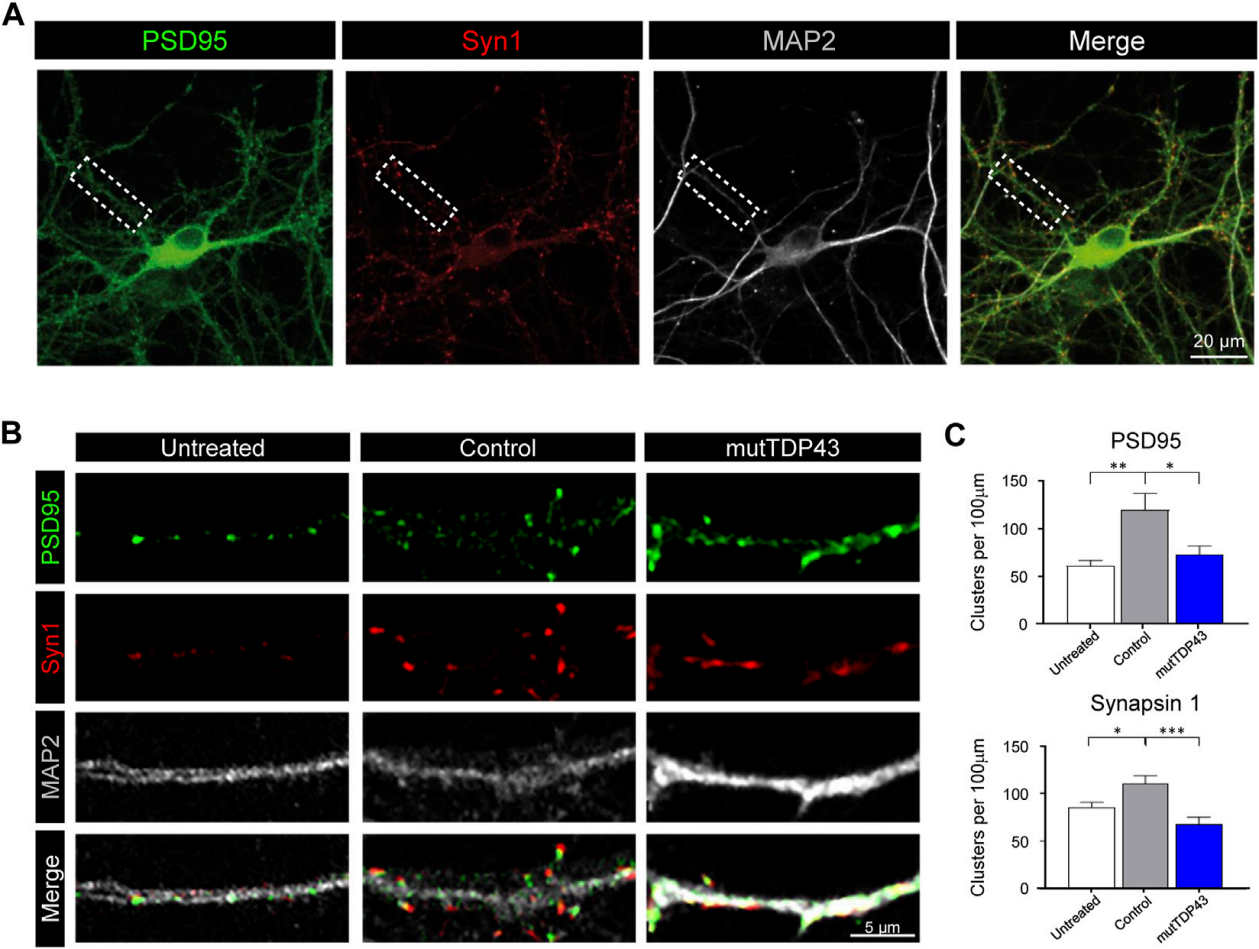
Mature mutTDP43 patient-derived astrocytes lose the ability to promote hippocampal excitatory synapse maturation. **(A)** Representative confocal images of an untreated 12 DIV hippocampal neurons with triple immunostaining to detect immunoreactive (IR) clusters of the postsynaptic anchoring protein PSD95 (green), the presynaptic marker synapsin-1 (Syn1; red) and the neuronal marker MAP2 (white), followed by the merge of the three markers. An example of a primary dendritic branch is indicated (dashed lined boxed). **(B)** Representative confocal images of primary dendritic branches of hippocampal neurons untreated or treated with ACM from control or mutTDP43 mature astrocytes. IR clusters for PSD95 (arrows) and synapsin-1 are detected in all conditions, and elevated in ACM from mature control subject-derived astrocytes, but not from mature mutTDP43 patient-derived astrocytes. **(C)** Quantifications are shown for the total number of PSD95-IR clusters (upper graph) and synapsin-1-IR clusters (lower graph) per 100 μm of dendritic branches of untreated neurons or treated neurons with ACM from control astrocytes or mutTDP43 astrocytes. Graphs show mean ± S.E.M. One-way ANOVA *p < 0.05, **p < 0.01, ***p < 0.001 control versus untreated and patient (*n* = three to five neurons per condition).

Healthy mouse and human astrocytes play critical roles during the development of the CNS and promote synapse maturation of diverse types of neurons (Ullian et al., 2001; Serio et al., 2013; Liddelow et al., 2017; Tchieu et al., 2019). Here, we investigated whether control subject- and mutTDP43 patient-derived astroglia maintain these functional capacities and hence are able to promote synaptogenesis of immature hippocampal neurons. For this, 7 days *in vitro* (DIV) mouse hippocampal neurons were treated with diluted astrocyte conditioned media (ACM) from mature control astrocytes (control-ACM) and mature mutTDP43 astrocytes (mutTDP43-ACM). As an additional control, neurons were kept untreated with any ACM and maintained in their culture media (untreated). At 12 DIV, cultures were fixed, and double immunofluorescence staining performed for detecting the post-synaptic anchoring protein PSD95 and the pre-synaptic marker synapsin-1. In agreement with our previous studies (Bustos et al., 2014; Ampuero et al., 2017), confocal images and their subsequent quantification analyses demonstrated that untreated 12 DIV hippocampal neurons exhibit discrete PSD95- and synapsin-1-immunoreactive (IR) clusters (Figure 3A–C). In agreement with the functional role of human iPSC-derived control subject astrocytes in neuronal co-cultures (Serio et al., 2013) and *in vivo* studies (Han et al., 2013), we also found that application of control-ACM to hippocampal neurons strongly increased the number of PSD95-IR and synapsin-1-IR clusters. The co-localization (or close opposition) of these pre- and post-synaptic markers further indicates the formation of structural synapses. In contrast, the number of PSD95-IR and synapsin-1-IR clusters remained unchanged in the presence of mutTDP43-ACM relative to untreated hippocampal cultures. These results indicate that while mature astrocytes derived from healthy control subjects potentiate excitatory synapse maturation, mutTDP43 patient-derived mature astrocytes lack this capacity.

### Mature mutTDP43 patient-derived astrocytes exhibit elevated intracellular polyP levels and secrete polyP that mediates MN death

**FIGURE 4:**
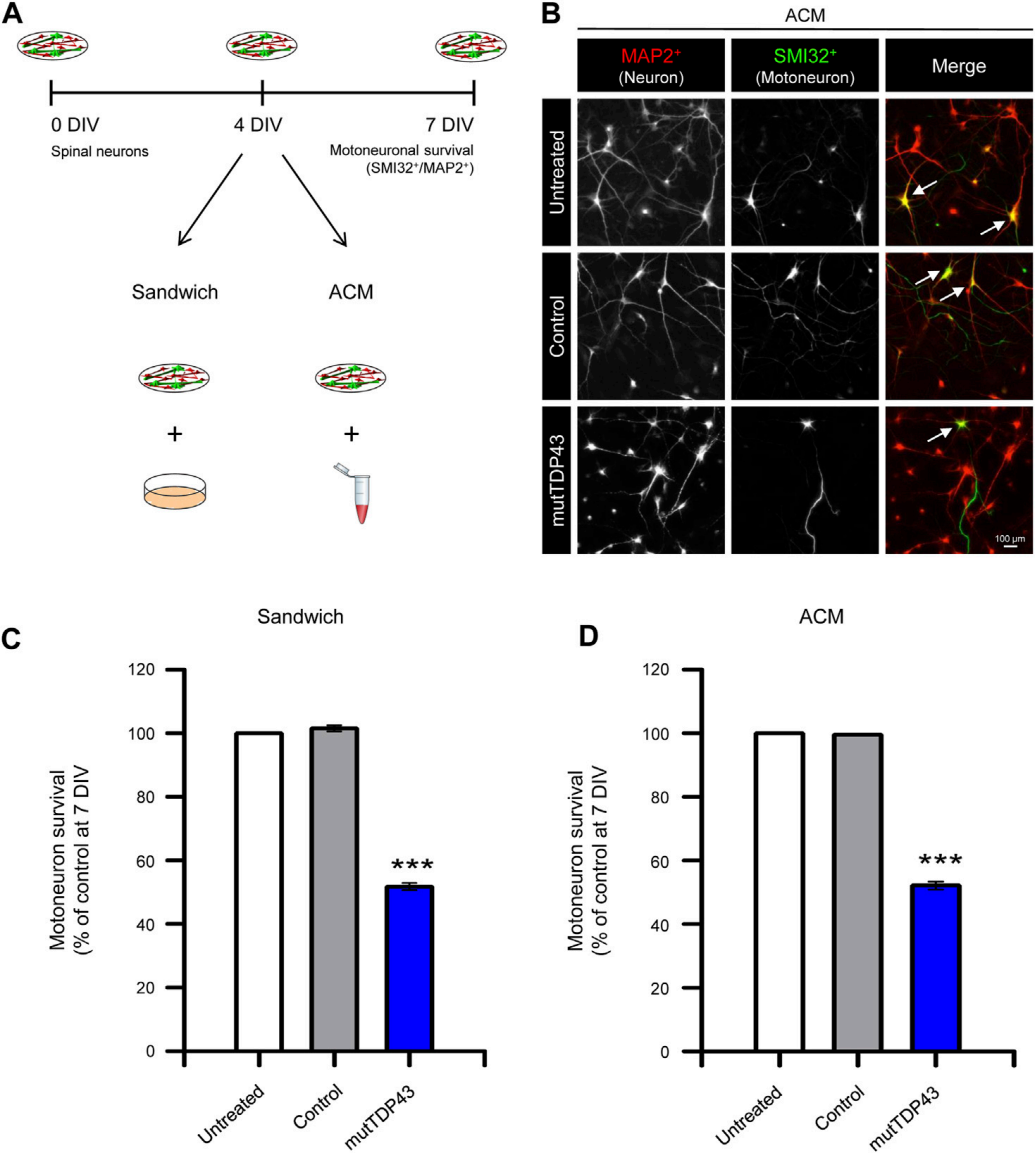
Mature mutTDP43 patient-derived astrocytes cause MN death in sandwich co-cultures or when treated with conditioned media. **(A)** Schematic of the study design to test primary MN survival in spinal cord cultures by using either a sandwich co-culture technique (Sandwich) with control or mutTDP43 mature astrocytes or by applying ACM from control or mutTDP43 mature astrocytes. Untreated spinal cord cultures were used as internal control. At 7 DIV, spinal cord cultures were fixed and neuronal survival was assayed by immunostaining with antibody against MAP2 to identify all neurons (interneurons plus MNs), and an antibody against SMI32 to selectively identify MNs. **(B)** Representative fluorescence images showing MAP2^+^/SMI32^+^ labeled neurons (white arrows) in the spinal cord cultures treated with the different ACMs as indicated. **(C–D)** Quantification of the percentage of MN survival (SMI32^+^/MAP2^+^ cells) in the **(C)** Sandwich and **(D)** ACM condition for spinal cord cultures either untreated or treated with control or mutTDP43 samples. Graphs show mean ± S.E.M. One-way ANOVA ***p < 0.001 patient versus untreated and control (*n* = 3 independent experiments).

**FIGURE 5:**
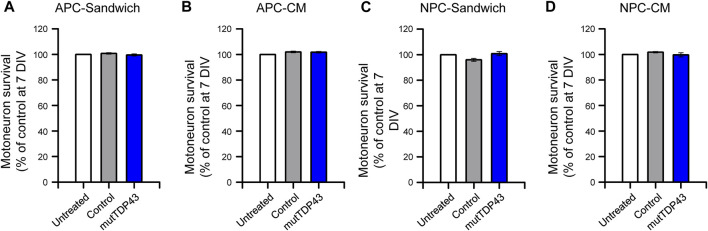
MN death is not caused by mutTDP43 astrocyte precursors or neuronal progenitors either in sandwich co-cultures or when treated with conditioned media. **(A–B)** Quantification of the percentage of MN survival in the **(A)** Sandwich condition with control or mutTDP43 astrocyte precursor cells (APCs) or **(B)** by applying conditioned media (CM) from control or mutTDP43 APCs. **(C–D)** Quantification of the percentage of MN survival in the **(C)** Sandwich condition with control or mutTDP43 neural progenitor cells (NPCs) or **(D)** by applying CM from control or mutTDP43 NPCs. For all experiments, untreated spinal cord cultures were used as internal control. Graphs show mean ± S.E.M. One-way ANOVA *p* > 0.05 patient versus untreated and control (*n* = 3 independent experiments).

We next determined whether human mutTDP43 mature astrocytes exhibit non-cell autonomous toxicity. For this purpose, we used primary spinal cord cultures either co-cultured with the human astrocytes (Sandwich) or treated with diluted ACM, starting at 4 DIV (Figure 4A). At 7 DIV, these spinal cord cultures were fixed and neuronal survival was assayed by double immunostaining against MAP2 to identify all neurons (interneurons plus MNs), and against SMI32 to selectively identify MNs (Figure 4B). It was found that both sandwich co-cultures (Figure 4C) or incubation with ACM (Figures 4B, D), show that mature mutTDP43 astrocytes, but not mature control subject astrocytes, caused extensive and specific MN death (40%–50%), without affecting the interneuron population (not shown). We also determined that the non-cell autonomous toxicity of mutTDP43 cells to MNs was dependent on their maturation stage; neither APCs (Figures 5A, B) nor NPCs (Figures 5C, D) reduced the number of MNs in sandwich co-cultures or after application of conditioned media.

### Mature mutTDP43 patient-derived astrocytes exhibit elevated intracellular and secreted polyP that mediates MN death

**FIGURE 6:**
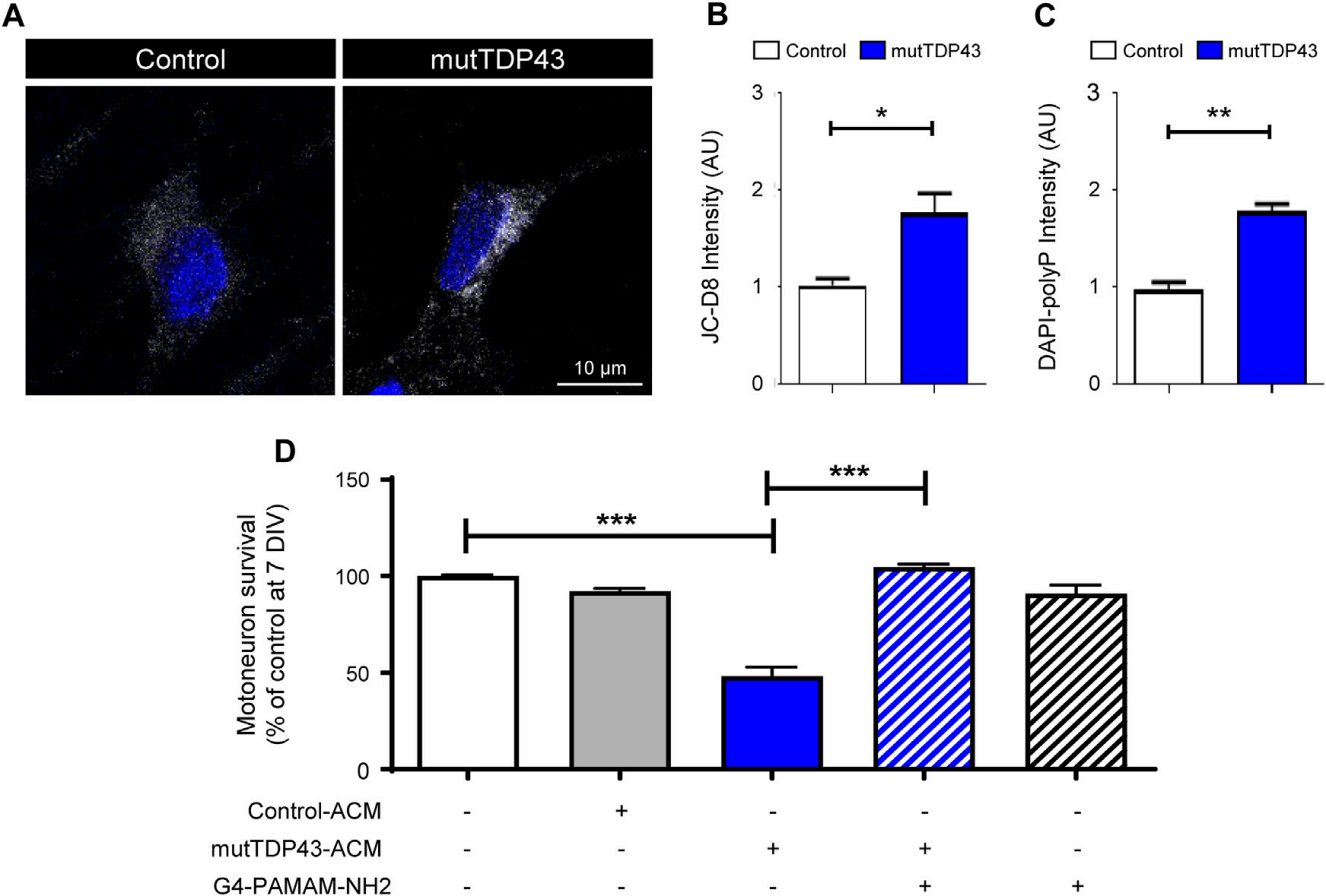
PolyP levels are elevated in mature mutTDP43 patient-derived astrocytes and neutralizing polyP in mutTDP43-ACM prevents MN death. **(A)** Confocal images of control and mutTDP43 mature astrocytes stained with JC-D8 (white) and TOPRO3 (blue) to detect polyP and nuclei, respectively. **(B–C)** Quantification of cytoplasmic polyP levels of individual human control and mutTDP43 mature astrocytes determined with **(B)** JC-D8 or **(C)** DAPI-polyP staining. Graphs show mean ± S.E.M. *p < 0.05, **p < 0.01; unpaired Student’s *t*-test mutTDP43 versus control (*n* = 3 independent experiment; ≥ 15–25 cells/condition). **(D)** Conditioned media from mature mutTDP43 astrocytes (mutTDP43-ACM), but not Control-ACM, causes extensive MN death. Application of G4-PAMAM-NH2 to mutTDP43-ACM prevents MN death. Graphs show mean ± S.E.M. of the average fluorescent intensity of the cell soma and expressed as arbitrary units (AU). ***p < 0.001; one-way ANOVA mutTDP43-ACM alone versus controls (control-ACM, mutTDP43-ACM + G4-PAMAM-NH2). Application of G4-PAMAM-NH2 alone does not alter MN survival (*n* = 3 independent experiments).

PolyP is enriched in human and mouse ALS/FTD astrocytes and its excessive release is toxic to MNs (Arredondo et al., 2022). We used two different commercially available dyes to detect polyP in human mutTDP43 astrocytes; JC-D8 (Ex: 488 nm; Em: 510–560 nm) and the less specific probe DAPI-polyP (Ex: 488 nm; Em: 510–560 nm) (Aschar-Sobbi et al., 2008; Angelova et al., 2014; Arredondo et al., 2022). In agreement with our previous studies using recombinant polyP-binding domain (termed recPPBD) to detect polyP (Arredondo et al., 2022), confocal images and subsequent quantification revealed that polyP staining with JC-D8 (Figures 6A, B; Supplementary Figure S4) and DAPI-polyP (Figure 6C) was ∼2-fold higher in mature mutTDP43 patient-derived astrocytes relative to control subject-derived astrocytes. To confirm that polyP is a critical toxic component secreted by mutTDP43 astrocytes, we investigated whether MN death can be prevented by neutralizing polyP in mutTDP43-ACM with G4-PAMAM-NH2, a dendrimer containing an ethylenediamine core and 64 positively charged primary -NH2 groups on the surface (14 kDa) and shown to efficiently bind and neutralize polyP (Smith et al., 2012; Travers et al., 2014; Arredondo et al., 2022). As shown in Figure 6D, it was found that MN death is prevented when G4-PAMAM-NH2 was added to mutTDP43-ACM.

## Discussion

Here we show an efficient, reproducible and scalable method to generate, under serum-free conditions, highly homogenous populations of mature astrocytes from control subject iPSCs and mutTDP43 patient iPSCs. These cultures were characterized based on analyzing multiple expression markers, indicatives of cell type, maturity and reactivity. Additionally, the functionality of our astrocyte cultures was analyzed by their ability to induce synaptogenesis of hippocampal neurons. We show that while functional quiescent mature astrocytes were generated from control subject-derived iPSCs, mature mutTDP43 patient-derived astrocytes displayed a mild reactive signature and were unable to promote synaptogenesis. These mutTDP43 astrocytes recapitulated a key aspect of TDP43-related ALS/FTD pathology by showing increased cytoplasmic TDP-43 levels, although without detectable TDP-43 aggregates or a significant reduction in nuclear TDP-43 signal. mutTDP43 mature astrocytes were also found to display elevated intracellular polyP levels and shown to excessively release polyP that is toxic to MNs. These results confirm our previous findings, indicating that an excessive release of polyP by mature ALS/FTD mutTDP43 astrocytes represents a critical component in non-cell-autonomous toxicity to MNs. The higher polyP staining in TDP43^A90V^ mature astrocytes are directly related to the elevated polyP-polymer concentration found in the ACM obtained from this patient’s astrocyte cultures and are in accordance with the increased polyP levels quantified in the cerebrospinal fluid (CSF) of a patient carrying a mutation in TDP43 (Arredondo et al., 2022). Given that these human TDP-43 mutant astrocytes were grown in the absence of significant numbers of neurons and microglia, our data suggest that the disease-dependent signature in these ALS/FTD astrocytes is cell-autonomous. We believe that the specific innate pathological signature of these ALS/FTD astrocytes opens important new avenues to decipher the molecular mechanisms underlying the generation of the neurotoxic phenotype.

We acknowledge the need for a cautious interpretation of the results obtained from a single patient in this study, and future investigations involving a broader range of patient samples would enhance the validity and generalizability of the findings. However, to address our research question, we have rigorously applied well-controlled methodologies, studying an iPSC line from an unaffected family member that serves as a negative control, thus minimizing genetic background disparities. Also, we have selected representative iPSC lines characterized in a previous investigation from our team (Zhang et al., 2013). In that study, we reported that three iPSC lines from an ALS/FTD patient with a TDP^*A90V*^ mutation and two lines derived from a family member can be differentiated to a similar extent to NPCs and subsequently to functional neurons (Zhang et al., 2013). Also, to determine common pathological features in diverse ALS/FTD patients, similar iPSC reprogramming and differentiation conditions were used to previously describe the generation of neurons from a patient harboring a TDP43^*M337V*^ mutation (Bilican et al., 2012). Unlike patient-derived TDP^*M337V*^ neurons, patient-derived TDP^*A90V*^ neurons displayed a tendency to accumulate TDP-43 in the cytoplasm but required cellular stress conditions to significantly increase cytoplasmic levels of TDP-43. Overexpression studies in HeLa cells reported that significant cytoplasmic accumulations of TDP-43 can be observed in cells ectopically expressing either TDP43^*M337V*^ or TDP43^*A90V*^. However, TDP43^*M337V*^ expression resulted in significantly more cellular parameters associated with the pathology including reduced TDP-43 protein solubility, increased levels of phosphorylated TDP43 (S109/S110), and the formation of C-terminal truncated protein fragments (Wobst et al., 2017). In addition to pathological differences between TDP43^*A90V*^ and TDP43^*M337V*^, patients carrying these mutations display distinctive clinical characteristics. Thus, while patients with the TDP43^*M337V*^ mutation developed ALS progression at an expected rate, subjects carrying TDP^*A90V*^ (including those from which the iPSCs were generated) experienced an exceptionally slow ALS/FTD progression following the initial diagnosis (Rutherford et al., 2008; Winton et al., 2008; Tamaoka et al., 2010; Zhang et al., 2013). Together, these studies indicate that the TDP43^*A90V*^ mutation is pathogenic but exhibits slower kinetics than the TDP43^*M337V*^ mutation, hence leading to a delayed disease progression. In summary, these findings are in agreement with our result showing a mild abnormal cytoplasmic localization of TDP-43 in mature TDP43^A90V^ patient-derived astrocytes. Our results are also in agreement with studies analyzing postmortem CNS samples of patients with TDP43 mutations (see below Discussion).

Our results with mutant *TARDBP* astrocytes are in agreement with a large number of previous studies showing that, independent of the genotype, fALS and fALS/FTD (*SOD1, TARDBP, C9ORF72* and *VCP*) as well as sALS astrocytes, can reduce the number of healthy wild-type MNs in co-cultures or after addition of their ACM to MN cultures (van Harten et al., 2021; Stoklund Dittlau and Van Den Bosch, 2023; and references included in the introduction). However, there are also reports using human iPSC-derived astrocytes that do not fully support this conclusion. Thus, a previous investigation using mutTDP43 astrocytes did not find a non-cell-autonomous toxic effect of these cells on MNs (Serio et al., 2013). On one hand, a potential explanation for these contradictory results can be related to the different mutation (TDP43^*M337V*^) that was studied, which unlike TDP43^*A90V*^, may not be capable of driving non-cell-autonomous processes. Alternatively, particular technical issues during the process of generating pathogenic astrocytes might also explain the differences between these human mutTDP43 glial cultures. Hence, albeit that in both investigations astrocyte cultures were produced under serum-free conditions, a different cocktail of growth factors was supplemented at the iPSC, NPC and APC stages to drive the maturation processes. This change was reflected in differences in the expression pattern of astrocytic gene markers at the final stage: elevated expression of S100β (as well as of EAAT2 and Cx43) accompanied by a reduced expression of GFAP in control and TDP43^*A90V*^ astrocytes versus enhanced expression of both S100β and GFAP in control and TDP43^*M337V*^ astrocytes. Based on the newly established criteria in the field, it has been suggested that this increased expression of GFAP detected in control and TDP43^*M337V*^ astrocytes (Serio et al., 2013) is indicative of a rather immature phenotype (Roybon et al., 2013). Recent studies using human iPSCs/iNPCs-derived astrocytes grown under serum-free conditions have also shown non-cell autonomous toxicity to MNs when these cells exhibit a strong S100β/ALDH1L1 immunostaining accompanied by an enhanced expression of GFAP in both control and sALS and fALS (*SOD1, TARDBP*, *C9ORF72* and *VCP*) astrocytes (Meyer et al., 2014; Hall et al., 2017; Birger et al., 2019; Varcianna et al., 2019; Zhao et al., 2020; Gomes et al., 2022). Taken together, these results indicate that under specific culture conditions the development of an immature (high GFAP) astrocytic phenotype may not be critically affecting the neurotoxic state of sALS and fALS human astrocytes carrying pathogenic gene mutations in *SOD1, TARDBP*, *C9ORF72* or *VCP*. It is tempting to speculate that in the case of astrocytes carrying mutations in the *TARDBP* gene, reaching a pathogenic phenotype that is capable of causing non-cell autonomous toxicity to MNs requires also the presence of particular parameters, including a mild reactivity state and the presence of pathogenic hallmarks.

TDP-43 is a predominantly nuclear protein, although it can shuttle between the nucleus and cytosol to exert multiple functions associated with RNA processing, including transcription, pre-mRNA splicing, trafficking and stabilization of mRNA (Tziortzouda et al., 2021; Brown et al., 2022; Halim and Gao, 2022; Baughn et al., 2023). Cytoplasmic TDP-43 aggregation in neurons is a prominent hallmark of ALS and FTD and often accompanied by depletion of TDP-43 nuclear levels (Neumann et al., 2006; Kawakami et al., 2019, but see below Wegorzewska et al., 2009). Mechanistic studies with ALS and FTD cell-based models indicate that disease-promoted cytoplasmic accumulations of TDP-43 can lead to the formation of TDP-43 aggregates that subsequently cause nuclear TDP-43 depletion and induce neuronal cell death (Walker et al., 2015; Gasset-Rosa et al., 2019; Tziortzouda et al., 2021). Studies with postmortem CNS samples from ALS and FTD patients also confirm that astrocytes exhibit TDP-43 aggregates in the cytoplasm, often paralleled by reduced nuclear TDP-43 levels (Geser et al., 2008; Gregory et al., 2020). Given that no mechanistic studies have yet been performed in astrocytes, it remains unclear whether TDP-43 dysregulation in ALS glia cells contributes to non-cell autonomous processes. Our data showing that iPSC-derived mature astrocytes harboring mutTDP43 exhibit only limited accumulation of TDP-43 in the cytoplasm indicate that neither cytoplasmic TDP-43 aggregates/inclusions nor its nuclear depletion is required for mutTDP43 non-cell autonomous toxicity to MNs. These results are in agreement with studies in the TDP43^*A315T*^ carrying transgenic mice where cytoplasmic TDP-43 aggregates in brain cells were not detected and nuclear loss of TDP-43 was only occasionally observed in neurons (Wegorzewska et al., 2009).

Finally, our study has potentially broad clinical projections. First, we previously showed that MN death can be prevented by degrading (using the yeast polyphosphatase PPX) or neutralizing (using G4-PAMAM-NH2 or UHRA9/10) polyP in diverse ALS/FTD-ACM (Arredondo et al., 2022). In agreement with these previous studies, we show with G4-PAMAM-NH2 that reducing polyP in ACM-mutTDP43 can be a therapeutic target of mutTDP43-mediated ALS/FTD. The versatility and reproducibility of PAMAM dendrimers, which efficiently bind and neutralize polyP but also other highly anionic linear polymers such as heparin (Smith et al., 2012), opens new avenues for the use of synthetic nano-sized tree-like branched polycationic compounds in ALS/FTD. However, in additional *in vivo* studies using high doses of PAMAM dendrimers to prevent thrombosis in a mouse model it has been found that these components can be toxic in mice (Roberts et al., 1996; Malik et al., 2000; Moreau et al., 2002). Recently, a new selective polyP inhibitor type was developed, termed macromolecular polyanion inhibitors (MPIs), that were shown to have an efficient anti-thrombotic activity in mouse models even at high doses (La et al., 2023). Second, additional studies with other subtypes of human ALS and ALS/FTD astrocytes are critical to determine whether iPSC-derived astrocytes will be useful in the future for personalized precision medicine testing and patient stratification. Third, iPSC-derived NPCs from healthy subjects have been gaining increasing support as potential sources for stem cell-based therapy development in ALS. Thus, it was recently shown that engraftment of human genetically engineered NPCs to deliver GDNF are capable of differentiating *in vivo* into astrocytes and protect spinal cord MNs in animal models (Baloh et al., 2023). Moreover, this study demonstrated that a single transplantation of these NPCs in human ALS patients provides new supporting cells that safely deliver GDNF to the ALS spinal cord; nevertheless, most grafted cells remained within the dorsal spinal cord. Our findings here that control NPCs are generated from iPSCs in an efficient, reproducible and scalable manner, which can be then differentiated to functional quiescent mature astrocytes make these NPCs interesting candidates for further evaluation of their potential in stem cell therapy studies.

## Data Availability

The original contributions presented in the study are included in the article/Supplementary Material, further inquiries can be directed to the corresponding authors.
