# Supplementary material for: Mature iPSC-derived astrocytes of an ALS/FTD patient carrying the TDP43 A90V mutation display a mild reactive state and release polyP toxic to motoneurons
Source: Front Cell Dev Biol. 2023 Jul 28;11:1226604. doi: 10.3389/fcell.2023.1226604 (PMC10461635; doi:10.3389/fcell.2023.1226604)
Supplement: Supplementary file 1 [file DataSheet1.PDF]

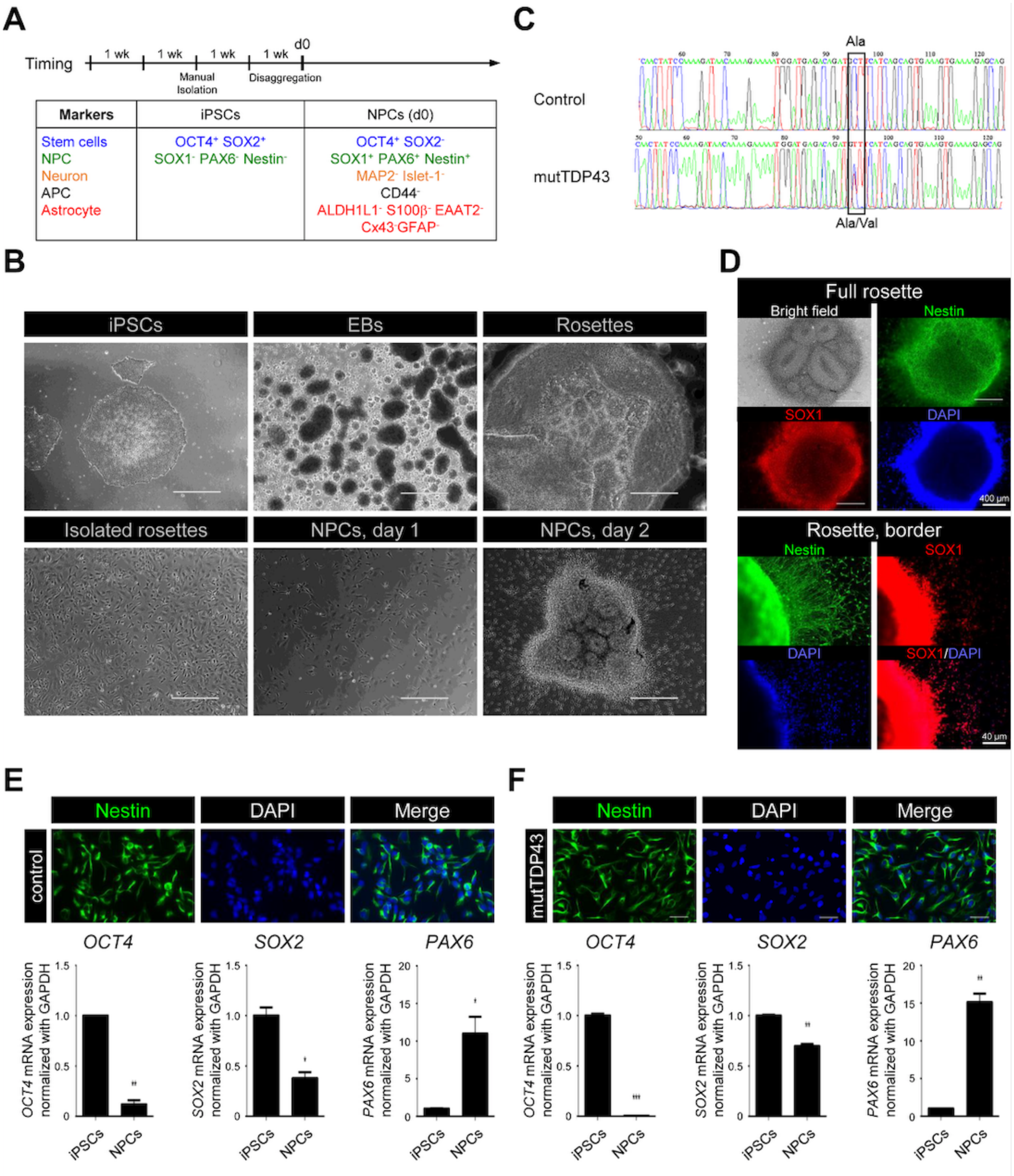

Supplementary Figure 1: Stem cell differentiation process to the neural lineage from control and TDP43 patient iPSCs.

(**A-B**) Overview and representative phase-contrast images show the differentiation process iPSCs (control subject and TDP43^{A90V} patient) generated by episomal transduction from skin biopsies were maintained in feeder-free conditions using mTeSR1 medium. EBs containing the three germ layers were generated and maintained in suspension for one week in EB differentiation media (KO-DMEM/F12 supplemented with KO serum replacement, Glutamax, NEAA, and 2-mercaptoethanol). To obtain neuroepithelial cells (rosettes), the EBs were plated in poly-

L-ornithine-laminin-coated plates and were growth for one week in neural induction media (KO-DMEM/F12 supplemented with N2, NEAA, heparin, and βFGF). Fully formed rosettes were manually isolated under the microscope, replated in matrigel-coated plates and then grown one more week in neural expansion media (neurobasal supplemented with Glutamax, NEAA, B-27, and βFGF). The disaggregation of rosettes using an accutase solution generated a stable, proliferative, monolayer culture of NPCs. (**C**) Expression of wild-type and A90V TDP43 transcript in control and $TDP43^{A90V}$ samples, respectively, was confirmed by RNA isolation, RT-PCR, cloning of amplicons into plasmids, and Sanger sequencing. (**D-F**) The identity of neural progenitor cells forming rosettes (panels **D1-D2**) or disaggregrated (**E-F**) was confirmed by immunofluorescence, using antibodies against Sox1 and Nestin antibodies or by RT-qPCR using specific Oct4, Sox2 and Pax6 primers. Scalebars: D1, 400 µm; D2-E-F: 40 µm. RT-qPCR results were obtained following the 2-ΔΔCT method and GAPDH was used as reference. Graphs show mean±S.D. Unpaired Student's *t*-test *$p<0.05$; **$p<0.01$; ***$p<0.001$ iPSCs *versus* NPCs (n=3 independent experiments).

A

Method	Media	Medium formulation	Reference	Poliferation capacity	Properties
1	*Astrocyte Alternative Medium*	Neurobasal, 5 ng/mL CNTF, 10 ng/mL BMP2, 8 ng/mL FGF, 1% FBS, 1x B27, 1x NEAA, 1x GlutaMAX, 50 ug/mL Penn-Strep	Shaltouki A, *et al.* Stem Cells 2013; 31:941-952		Medium proliferation capacity. Generates CD44+ cells by d14 Cells stop proliferating at d28 Low GFAP+ after d28.
**2**	**Astrocyte Precursor Medium**	KO DMEM/F12, 1x StemPro NSCs Supplement, 10 ng/mL Activin A, 10 ng/mL Heregulin 1β, 200 ng/mL IGF1, 20 ng/mL FGF, 20 ng/mL EGF, 1x GlutaMAX	Shaltouki A, *et al.* Stem Cells 2013; 31:941-952		High proliferation capacity. Generates CD44+ cells by d14 **Cells proliferate for at least 6 wks.** Low GFAP+ after d28
3	*Astrocyte Alternative Medium*	Neurobasal, 100 nM TSA (1st 48 h), 500 nM 5-azaC (1st 48 h), 20 ng/mL BMP2, 1x B27, 1x GlutaMAX, 50 ug/mL Penn-Strep, 10 ng/mL LIF	Majumder A, *et al.* Stem Cell Research 2013; 11:574-586		Does not produce high CD44+ nor GFAP+ populations.

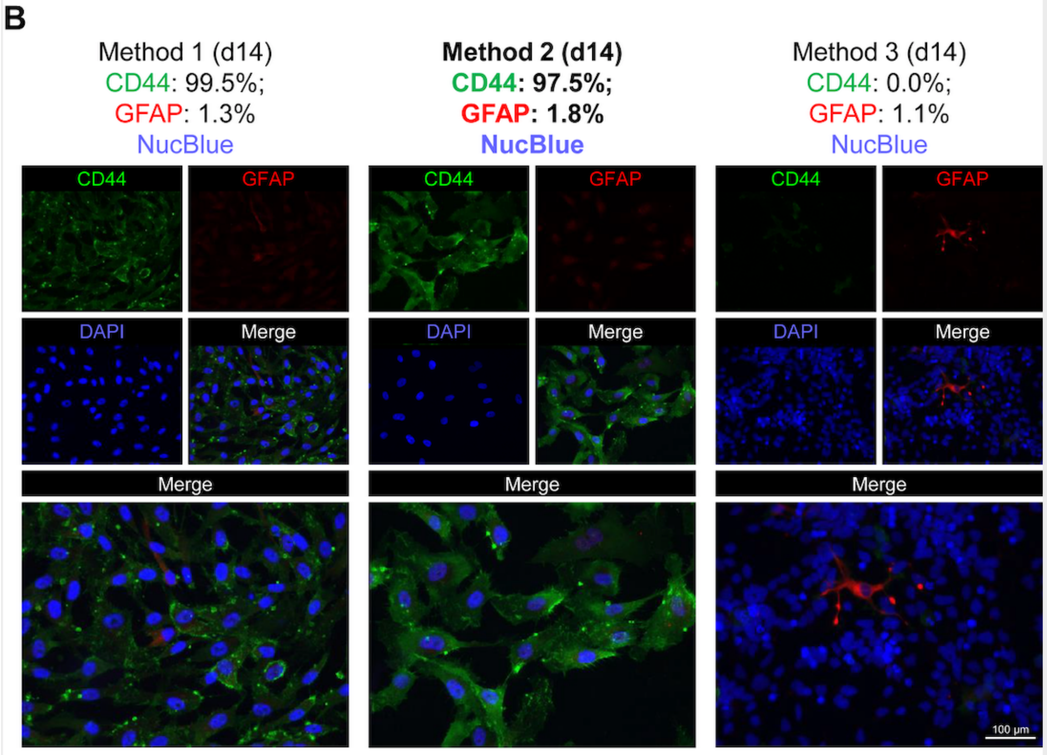

Supplementary Figure 2. Generation of CD44$^+$ APCs from control NPCs.
Table showing the methods 1, 2 and 3 -their specific media formulations and references- used for 2 weeks to trigger astrocytic differentiation from NPCs. Representative confocal images showing immunofluorescence staining using antibodies against CD44 and GFAP. Media 1 and 2 generated proliferating CD44+ cells after 14 days of treatment (d14), while medium 3 was less efficient and astrocytic cells stopped proliferating under this condition. A low percentage of GFAP+ cells was found in all conditions. Particularly with method 2, an uniform scalable population of healthy proliferating CD44+ cells was obtained and this method was selected in the final protocol (Figure 1A).

A

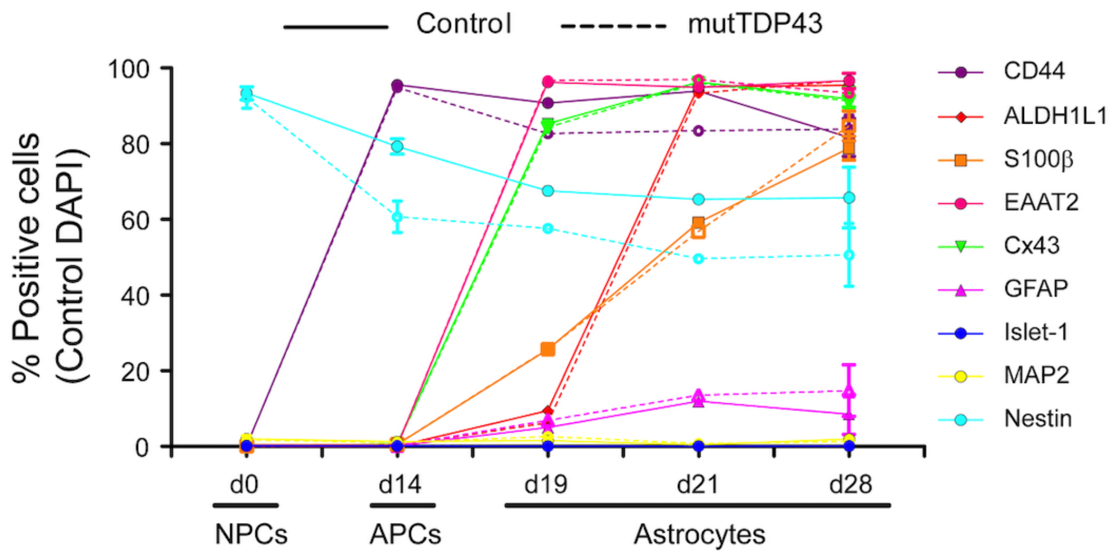

B

Mark	Type	d0	d14	d28
		Mean ± SE (n = 3)	Mean ± SE (n = 3)	Mean ± SE (n = 3)
**CD44**	Control	0.41 ± 0.1	95.5 ± 0.5	81.64 ± 5
	Patient	0.12 ± 0.1	94.77 ± 0.5	83.81 ± 4
**ALDH1L1**	Control	0.31 ± 0.2	0 ± 0	95.36 ± 0.7
	Patient	0.39 ± 0.2	0.19 ± 0.2	96.68 ± 0.3
**S100β**	Control	0.34 ± 0.3	0.42 ± 0.3	78.82 ± 3
	Patient	0.15 ± 0.1	0.5 ± 0.2	84.65 ± 4
**EAAT2**	Control	0 ± 0	0 ± 0	96.59 ± 2
	Patient	0 ± 0	0 ± 0	93.26 ± 0.9
**Cx43**	Control	0 ± 0	0.61 ± 0.6	91.75 ± 3
	Patient	0 ± 0	0.48 ± 0.5	91.19 ± 2
**GFAP**	Control	0 ± 0	0.11 ± 0.1	8.42 ± 5
	Patient	0 ± 0	0.24 ± 0.2	14.68 ± 7
**Islet-1**	Control	0.19 ± 0.2	0.15 ± 0.2	0.12 ± 0.1
	Patient	0.16 ± 0.2	0.21 ± 0.2	0.15 ± 0.2
**MAP2**	Control	1.96 ± 0.9	1.2 ± 0.8	1.9 ± 0.3
	Patient	1.62 ± 1.0	0.98 ± 0.7	1.19 ± 0.2
**Nestin**	Control	93.28 ± 2	79.19 ± 2	65.73 ± 8
	Patient	92.14 ± 3	60.63 ± 4	50.55 ± 8

Supplementary Figure 3. Characterizations of control and mutTDP43 patient NPCs, APCs and mature astrocytes.

Graph and table extend the quantification data shown in Figure 1. Quantification of the percentage of positive cells for the different markers for control and mutTDP43 patient samples at d0, d14, d19, d21 and d28. Immunostaining assays was performed with antibodies against specific canonical markers for key developmental stages, including NPCs (Nestin), APCs (CD44), astrocytes (ALDH1L1, S100β, EAAT2, Cx43 and GFAP) and neurons (MAP2, Islet-1). Data was obtained for 3 independent differentiations at d0, d14 and d28. At least 15 cells analyzed per condition were analyzed in each experiment. Student's *t*-test analysis revealed no significant difference (p>0.05) between control and patient samples for any NPC, APC or mature astrocyte cultures at any given time point.

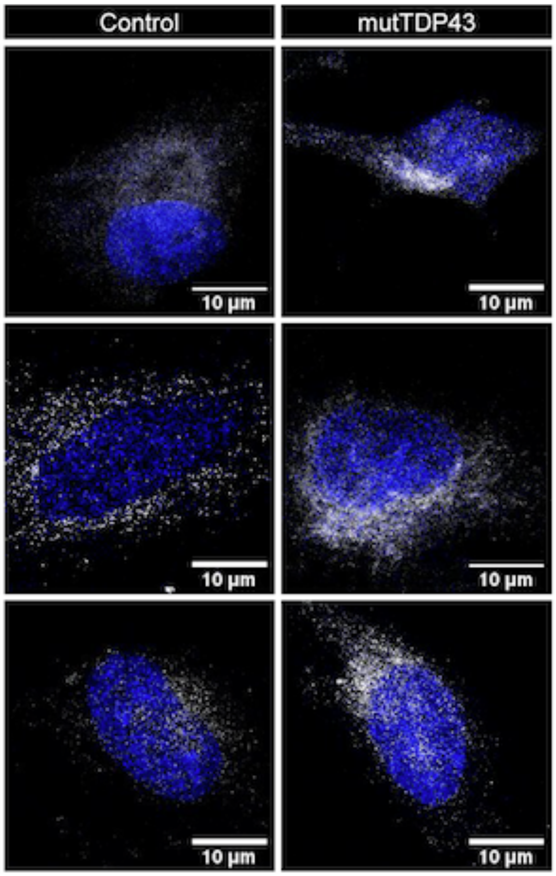

Supplementary Figure 4. PolyP levels are elevated in mature mutTDP43 patient-derived astrocytes. Confocal images of additional control and mutTDP43 mature astrocytes stained with JC-D8 (white) and TOPRO3 (blue) to detect polyP and nuclei, respectively.
